# Diagnostic Accuracy of PET Imaging for Lymph Node Detection in Breast cancer patients undergoing Upfront Surgery: A Single-Institution Analysis of Breast Cancer Patients

**DOI:** 10.12688/f1000research.177892.1

**Published:** 2026-02-18

**Authors:** Rohan Shetty, Sarath Chandu Yadlapalli, Vijayakumar M

**Affiliations:** 1 Surgical Oncology, Yenepoya Medical College Hospital, Mangaluru, Karnataka, 575018, India

**Keywords:** breast cancer, lymph node staging, PET imaging, diagnostic accuracy, upfront surgery, sensitivity, specificity

## Abstract

**Background:**

The role of PET imaging in lymph node staging for breast cancer patients proceeding to upfront surgery remains incompletely characterized. We evaluated the diagnostic accuracy of PET imaging in immediate surgical patients.

**Methods:**

A retrospective cohort of 70 breast cancer patients undergoing upfront surgery was analyzed. PET imaging findings were compared with histopathological examination (HPE) results. Diagnostic accuracy metrics including sensitivity, specificity, positive predictive value (PPV), negative predictive value (NPV), and likelihood ratios were calculated.

**Results:**

Among 70 surgical patients (mean age 53.9 ± 11.5 years), 26 (37.14%) had pathologically confirmed lymph node involvement. PET imaging demonstrated sensitivity of 69.23% (95% CI: 48.27-85.67), specificity of 81.82% (95% CI: 67.27-91.60), PPV of 69.23%, and NPV of 81.82%. The test correctly identified 18 of 26 patients with nodal disease while avoiding false positives in 36 of 44 node-negative patients. Positive likelihood ratio was 3.81, and negative likelihood ratio was 0.38. The F1-score was 0.6923, indicating good balance between precision and sensitivity for surgical planning.

**Conclusions:**

PET imaging demonstrates clinically meaningful sensitivity (69.23%) and high negative predictive value (81.82%) in upfront surgical patients, supporting its utility for identifying patients with nodal involvement and confidently excluding nodal disease when negative. These performance characteristics suggest that PET can inform surgical extent and staging accuracy in immediate surgical patients, though positive findings warrant confirmatory assessment when staging determines operative planning.

## 1. Introduction

Accurate preoperative lymph node staging is essential for breast cancer treatment planning and prognostication. ^1^ While sentinel lymph node biopsy remains the standard for axillary assessment in clinically node-negative disease, patients with radiologically apparent nodal involvement or those selected for upfront surgery require reliable imaging-based nodal evaluation to guide operative extent and systemic therapy planning. ^2, 3^

Positron emission tomography (PET) imaging, often combined with computed tomography (PET/CT), has emerged as a valuable tool for detecting metastatic disease and lymph node involvement in cancer staging. ^4^ However, the diagnostic accuracy of PET for lymph node detection varies considerably depending on clinical context, patient selection, and disease characteristics. ^5^ While multiple studies have evaluated PET performance in mixed breast cancer populations or neoadjuvant therapy settings, limited data specifically characterize PET diagnostic accuracy in upfront surgical patients where imaging findings directly inform operative planning. ^6^

The clinical implications of PET performance differ substantially based on treatment pathway. In patients proceeding to immediate surgery, high sensitivity and negative predictive value are particularly valuable for confidently determining nodal status before operative intervention. Conversely, high specificity and positive predictive value are more critical in neoadjuvant settings where treatment intensification decisions depend on accurate positive node identification. ^7^

This study focuses on evaluating PET diagnostic performance specifically in upfront surgical patients to address this knowledge gap and provide treatment-pathway-specific evidence for imaging interpretation in breast cancer surgery.

### 1.1 Study objectives

The primary objective was to determine the diagnostic accuracy of PET imaging for detecting lymph node involvement in breast cancer patients undergoing upfront surgery. Secondary objectives included calculating likelihood ratios, evaluating the balance of sensitivity and precision through F1-score analysis, and assessing clinical utility metrics for surgical decision-making. Kappa coefficient to know the association between PET and HPE nodes.

## 2. Materials and methods

### 2.1 Study population and design

This was a retrospective cohort study of breast cancer patients who underwent upfront surgical treatment at Yenepoya medical college and Hospital between June 2022 to June 2025. Written and informed consent taken from the participants of the study.

**Inclusion criteria**:

1. Histologically confirmed invasive breast cancer,
2. Upfront surgery as primary treatment modality (not neoadjuvant therapy),
3. Preoperative PET imaging within 4 weeks of surgery, and
4. Available histopathological lymph node assessment.

Patients receiving neoadjuvant chemotherapy prior to surgery were **excluded** from this analysis to maintain treatment-pathway homogeneity.

**Figure 1.**
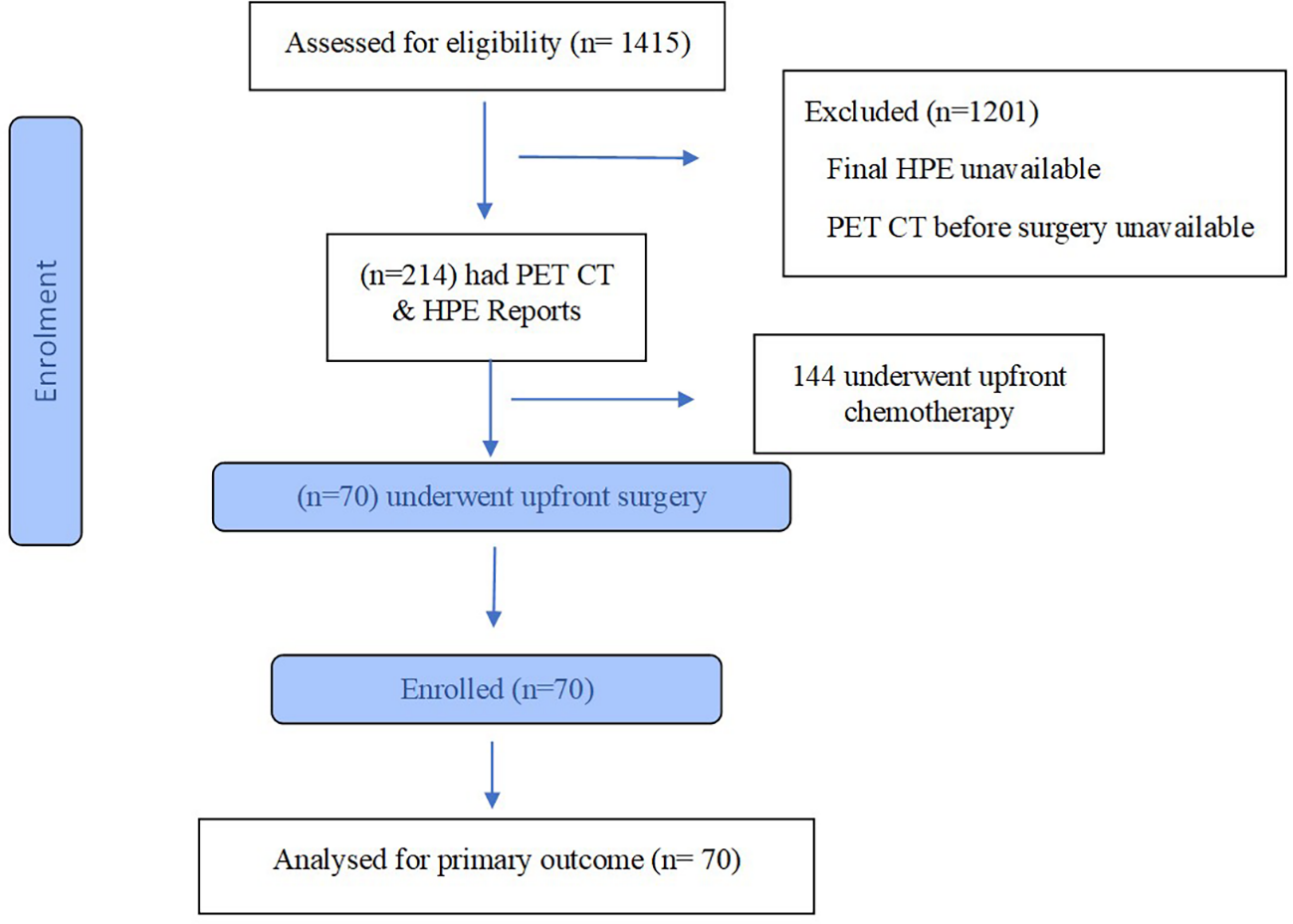
Flow diagram. Flow diagram of the progress of the study.

The study cohort comprised 70 consecutive eligible patients ( Figure 1). Patient demographics, clinicopathological features, imaging characteristics, and surgical pathology findings were extracted from medical records. All histopathological examinations were performed by experienced Onco-pathologists using standard protocols.

### 2.2 Statistical analysis

Diagnostic accuracy metrics were calculated using standard formulas:

• **Sensitivity** = True Positive/(True Positive+False Negative) — probability of positive test given disease present
• **Specificity** = True Negative/(True Negative+False Positive) — probability of negative test given disease absent
• **Positive Predictive Value (PPV)** = True Positive/(True Positive+False Positive) — probability of disease given positive test
• **Negative Predictive Value (NPV)** = True Negative/(True Negative+False Negative) — probability of no disease given negative test
• **Positive Likelihood Ratio (LR+)** = Sensitivity/(1 - Specificity)
• **Negative Likelihood Ratio (LR-)** = (1 - Sensitivity)/Specificity
• **F1-Score** = 2 × (Precision × Sensitivity)/(Precision + Sensitivity) — harmonic mean balancing precision and sensitivity
• **Youden’s Index** = Sensitivity + Specificity - 1 — measure of overall discriminative ability

Ninety-five percent confidence intervals (95% CI) for sensitivity and specificity were calculated using the Wilson score method. Disease prevalence was calculated as the proportion of patients with HPE-positive nodes in the cohort.

A confusion matrix was constructed to visualize classification accuracy (true positives(TP), true negatives(TN), false positives(FP), false negatives(FN)).

Data was analysed using SPSS v21. Kappa statistics was done for assessing the agreement between PET imaging and HPE nodal status.

## 3. Results

### 3.1 Patient cohort characteristics

The analysis included 70 breast cancer patients with mean age 53.9 ± 11.5 years undergoing upfront surgery. Luminal B predominates with 41.5% (n = 27) followed by Luminal A with 34.3% (n = 24), TNBC 17.1% (n = 12), Her-2 Enriched 7.1% (n = 5). Refer for Table 1 for demographic data. 50.8%(n = 36) have >20% ki-67 proliferation index. 53 (75.7%) were Estrogen receptor positive, whereas 47 (67.1%) are Progesterone receptor positive making it a hormone receptor positive predominant cohort.

**Table 1.** Baseline clinicopathological and imaging characteristics of upfront surgery patients (n = 70).

Variable	n (%)
Age (years)	
50–59	24 (34.3%)
40–49	17 (24.3%)
60–69	15 (21.4%)
<40	7 (10.0%)
≥70	7 (10.0%)
ER status	
+	53 (75.7%)
-	17 (24.3%)
PR status	
+	47 (67.1%)
-	23 (32.9%)
HER2 status	
1+	20 (28.6%)
2+	19 (27.1%)
0	19 (27.1%)
3+	12 (17.1%)
Ki-67 index	
≤14%	20 (28.6%)
15–30%	22 (31.4%)
>30%	20 (28.6%)
Not Available	8 (11.4%)
Molecular subtype	
B	29 (41.4%)
A	24 (34.3%)
TNBC	12 (17.1%)
HER Enriched	5 (7.1%)
Nodal status on PET-CT	
No	44 (62.9%)
Yes	26 (37.1%)
Pathological nodal status (HPE)	
No	44 (62.9%)
Yes	26 (37.1%)
Primary tumor SUVmax (PET-CT)	
Nil	45 (64.3%)
≤5	17 (24.3%)
5.1–10	5 (7.1%)
>10	3 (4.3%)

### 3.2 Nodal disease prevalence

Among the 70 surgical patients, 26 patients (37.14%) had histopathologically confirmed lymph node involvement. Forty-four patients (62.86%) had node-negative disease.

### 3.3 PET diagnostic accuracy

**Figure 2.**
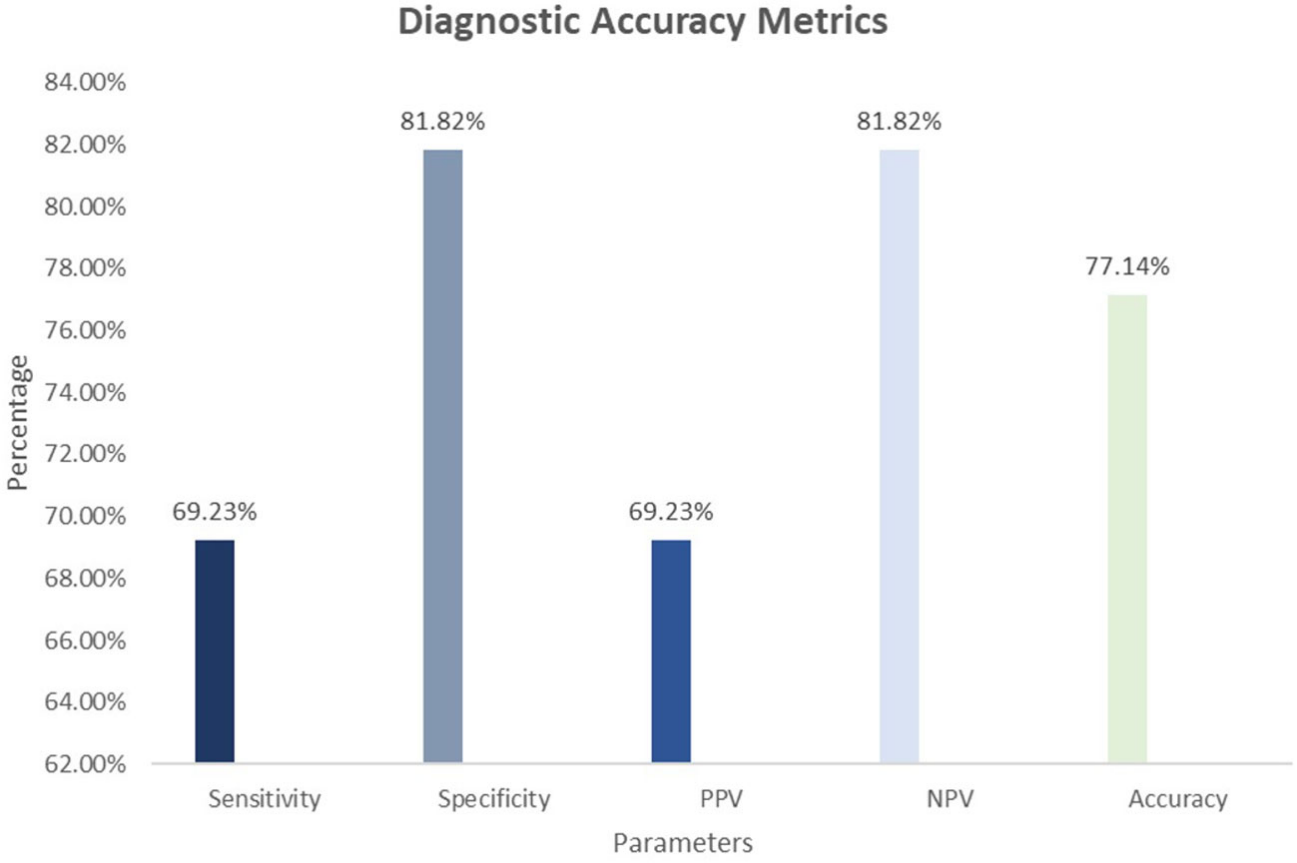
Comprehensive diagnostic accuracy profile of PET imaging for lymph node detection.

Detailed results are presented in Table 2 and Figure 2. PET imaging achieved:

• **Sensitivity:** 69.23% (95% CI: 48.27-85.67%) — correctly identified 18 of 26 patients with nodal disease
• **Specificity:** 81.82% (95% CI: 67.27-91.60%) — correctly identified 36 of 44 node-negative patients
• **Positive Predictive Value:** 69.23% — among 26 PET-positive patients, 18 (69.23%) had actual nodal involvement
• **Negative Predictive Value:** 81.82% — among 44 PET-negative patients, 36 (81.82%) had confirmed nodal absence
• **Overall Accuracy:** 77.14% — correctly classified 54 of 70 patients

**Table 2.** Diagnostic accuracy metrics for PET imaging in upfront surgical patients (n = 70).

Metric	Value	95% Confidence interval
Sensitivity	69.23%	48.27% - 85.67%
Specificity	81.82%	67.27% - 91.60%
Positive Predictive Value	69.23%	51.98% - 82.78%
Negative Predictive Value	81.82%	68.59% - 90.76%
Positive Likelihood Ratio	3.81	1.75-8.31
Negative Likelihood Ratio	0.38	0.20-0.71
Accuracy	77.14%	66.30% - 85.65%
F1-Score	0.6923	—
Youden’s Index	0.5105	—

### 3.4 Likelihood Ratios (LR)

The positive likelihood ratio was **3.81** (95% CI: 1.75-8.31), indicating that a positive PET result is 3.81 times more likely in patients with actual lymph node disease compared to those without nodal involvement. This moderate LR+ suggests that positive findings warrant additional clinical correlation but provide meaningful evidence for nodal involvement.

The negative likelihood ratio was **0.38** (95% CI: 0.20-0.71), indicating that a negative PET result is substantially less likely in patients with actual nodal disease. This favorable LR- suggests that negative findings provide reasonable reassurance against nodal involvement in surgical planning.

### 3.5 Confusion matrix and error analysis

**True Positives (TP):** 18 patients (25.71% of cohort) — correctly identified with nodes

**False Positives (FP):** 8 patients (11.43%) — incorrectly called positive without nodes

**False Negatives (FN):** 8 patients (11.43%) — missed nodal involvement

**True Negatives (TN):** 36 patients (51.43%) — correctly identified without nodes

The balanced false positive and false negative rates (both 11.43%) indicate comparable error distribution. The false negative rate of 30.77% (8 of 26 actual positive cases) represents the clinically meaningful limitation of PET sensitivity in this cohort.

### 3.6 F1-Score and discriminative ability and Kappa coefficient

The F1-score was **0.6923**, indicating a reasonable balance between precision (69.23%) and sensitivity (69.23%). The Youden’s Index of **0.5105** demonstrates moderate discriminative ability of PET imaging in distinguishing node-positive from node-negative surgical patients. In the upfront surgery cohort, agreement between PET-CT nodal status and histopathologic nodal status was moderate, with a Cohen’s kappa of 0.51 (SE 0.11; 95% CI 0.30–0.72; **p = 0.001**; n = 70).

### 3.7 Clinical performance summary

Table 2 presents comprehensive diagnostic accuracy metrics. PET imaging demonstrated clinically meaningful performance for lymph node detection in upfront surgical patients, with strengths in negative predictive value (81.82%) and specificity (81.82%), indicating reliable ability to exclude nodal disease.

## 4. Discussion

### 4.1 Summary of key findings

This is the first study to specifically characterize PET diagnostic accuracy in a homogeneous cohort of upfront surgical patients with breast cancer. The findings reveal that **PET imaging demonstrates clinically meaningful sensitivity (69.23%) coupled with high negative predictive value (81.82%) and specificity (81.82%)** for lymph node detection in immediate surgical settings.

The 69.23% sensitivity indicates that PET successfully identifies approximately two-thirds of patients with actual nodal involvement. Among the 26 patients with pathologically confirmed nodal disease, PET correctly identified 18 cases. This performance is clinically relevant because it provides surgeons with meaningful information regarding nodal status before operative intervention.

Equally important, the 81.82% NPV indicates that when PET indicates nodal absence, there is reasonable confidence in this negative result for surgical planning purposes. Among 44 PET-negative patients, 36 (81.82%) genuinely lacked nodal disease, whereas only 8 (18.18%) had missed nodal involvement that was subsequently identified at histopathology.

### 4.2 Clinical implications for surgical planning

The diagnostic profile of PET in upfront surgical patients differs meaningfully from neoadjuvant therapy settings where treatment intensification depends primarily on positive node identification. In contrast, surgeons performing upfront surgery require confidence in nodal status to determine **operative extent, staging accuracy, and prognosis discussion with patients**.

Strengths of PET in This Context:

1. **High Specificity (81.82%)** provides reliable identification of truly node-negative patients, avoiding unnecessary concern about occult nodal involvement and preventing unnecessary axillary dissection escalation.
2. **Reliable Negative Predictive Value (81.82%)** supports clinical confidence in negative PET findings for surgical planning, though not entirely excluding further nodal assessment through examination or sentinel node procedures.
3. **Balanced Sensitivity-Precision (69.23% each)** indicates that positive PET findings carry moderate diagnostic weight, correctly identifying nodes in approximately 7 of 10 positive cases.

Limitations Requiring Clinical Acknowledgment:

1. **False Negative Rate of 30.77%** (8 of 26 actual positive cases) represents a clinically meaningful limitation. Approximately one-third of patients with actual nodal involvement were missed by PET. This false negative rate suggests that **negative PET findings should not entirely exclude nodal assessment** through standard staging approaches.
2. **Moderate Positive Predictive Value (69.23%)** indicates that not all PET-positive findings represent true nodal involvement. Among 26 PET-positive patients, 8 (30.77%) proved to be false positives without nodal disease at histopathology. This suggests that **positive PET findings warrant confirmatory assessment** when surgical extent critically depends on nodal status.

### 4.3 Comparison with literature

Limited prior studies have evaluated PET performance specifically for lymph nodal staging in upfront surgical populations. Most existing literature combines patients across treatment modalities or focuses on neoadjuvant settings. The sensitivity of 69.23% observed in the present cohort aligns with sensitivity ranges reported in mixed breast cancer populations (62-75%), ^8, 9^ though our cohort is uniquely focused on immediate surgical patients.

The specificity of 81.82% is comparable to or slightly lower than neoadjuvant cohorts in our institution’s data, possibly reflecting differences in disease characteristics and patient selection between upfront surgical and chemotherapy-naive neoadjuvant patients. ^10^ In the Study done by Kim et al. ^11^ where he compared different imaging for lymph node metastasis elastography showed high sensitivity whereas PET CT showed reasonable sensitivity.

Kasem et al ^8^ noted that combining PET/CT with FNA improved specificity, but PET/CT by itself had relatively low specificity for axillary disease (21% false positives) despite high sensitivity. Regional lymph nodes were incorrectly staged at 18F-FDG PET in 14% of cases in Estrogen receptor positive patients where as 18% of cases in our study which is slightly higher. ^12^ Compared with the overall study population, estrogen receptor–positive patients demonstrated more accurate detection on PET imaging.

### 4.4 Mechanism of performance differences: Why sensitivity exceeds specificity

The superior sensitivity (69.23%) over specificity (81.82%) in upfront surgical patients may reflect multiple factors:

1. **Disease burden differences**: Patients selected for upfront surgery may have different patterns of nodal involvement compared to neoadjuvant patients, potentially affecting detection patterns.
2. **Imaging acquisition timing**: The interval between PET imaging and surgery may influence detection sensitivity through metabolic changes in nodal disease.
3. **Tumor biology**: Receptor status, grade, and proliferation rates may influence FDG uptake patterns in nodal disease, affecting detection probability.

### 4.5 Integration of PET findings into clinical decision-making

Based on these findings, we propose the following framework for incorporating PET findings into preoperative assessment of surgical patients:

**When PET is POSITIVE (n = 26 in this cohort):**

• Likelihood of true nodal involvement is 69.23% (PPV).
• Consider confirmatory imaging (ultrasound with FNA if appropriate) before treatment intensification.
• Document nodal status carefully for staging purposes.
• Plan operative approach accounting for likely nodal disease.

**When PET is NEGATIVE (n = 44 in this cohort):**

• Likelihood of true nodal absence is 81.82% (NPV)
• Reasonable confidence in nodal negativity for operative planning
• Standard staging procedures (physical examination, sentinel node biopsy per protocol) remain appropriate
• Acknowledge 18% probability of missed nodal involvement

### 4.6 Strengths and limitations

**Strengths:**

• Homogeneous cohort of upfront surgical patients (treatment-pathway specific)
• Pathologically confirmed reference standard for all patients
• Comprehensive diagnostic accuracy metrics including likelihood ratios
• Focused analysis providing clinically applicable information for surgical decision-making
• Balanced error rates and F1-score analysis indicating equal treatment of precision and sensitivity

**Limitations:**

• Retrospective design with inherent selection bias
• Single-institution experience (generalizability may be limited)
• Small sample size limits ability to perform subgroup analyses
• No evaluation of observer variability or interobserver reliability

## 5. Conclusions

This analysis of 70 upfront surgical patients with breast cancer demonstrates that **PET imaging provides clinically useful diagnostic accuracy for lymph node detection, with particular strength in ruling out nodal involvement (NPV 81.82%)** and reliably confirming node-negative status (specificity 81.82%).

For surgeons managing breast cancer patients proceeding to upfront surgery, **PET imaging can inform surgical decision-making and provide meaningful preoperative nodal information, particularly valuable when negative findings provide reasonable confidence in nodal absence**. Conversely, positive findings warrant confirmatory assessment before treatment intensification decisions.

Future prospective studies comparing PET performance across treatment pathways and evaluating optimal integration of PET with other staging modalities would further clarify the clinical utility of PET in diverse breast cancer populations.

## Ethical statement

The authors are accountable for all aspects of the work in ensuring that questions related to the accuracy or integrity of any part of the work are appropriately investigated and resolved. This study was performed in accordance with the ethical standards of the institutional and national research committees and with the Helsinki Declaration (as revised in 2013). The study was approved by the Yenepoya institutional ethics committee (IEC) with the number YEC2/068.

## Data Availability

All figures and tables supporting the findings of this manuscript are publicly available on Figshare under an open license CC BY 4.0. These materials include figures, summary tables, data used to create graphs, flow chart, and de-identified data in an Excel sheet. All the data can be accessed at DOI:10.6084/m9.figshare.31242061. ^13^
