# Supplementary material for: Diagnostic Accuracy of PET Imaging for Lymph Node Detection in Breast cancer patients undergoing Upfront Surgery: A Single-Institution Analysis of Breast Cancer Patients
Source: F1000Res. 2026 Jun 18;15:291. Originally published 2026 Feb 18. [Version 3] doi: 10.12688/f1000research.177892.3 (PMC13294660; doi:10.12688/f1000research.177892.3)
Supplement: Peer review history

# Reviewer report: Reviewer response for version 3

**DOI:** 10.5256/f1000research.203681.r494988

**Published:** 2026-06-24

**Authors:** Mehmet Tarık Tatoğlu

**Affiliations:** 1 Göztepe Prof. Dr. Süleyman Yalçin City Hospital, Istanbul, Turkey

The authors have addressed my remaining concern regarding the wording of the abstract conclusion. The revised abstract now appropriately reflects the moderate sensitivity of PET/CT and emphasizes that negative findings should be interpreted in the context of overall clinical, radiological, and pathological assessment.

 I appreciate the authors’ careful and constructive revisions throughout the review process. The manuscript has improved substantially and is now suitable for approval.

 I therefore update my recommendation to Approved.

Is the work clearly and accurately presented and does it cite the current literature?

Partly

If applicable, is the statistical analysis and its interpretation appropriate?

Partly

Are all the source data underlying the results available to ensure full reproducibility?

Partly

Is the study design appropriate and is the work technically sound?

Partly

Are the conclusions drawn adequately supported by the results?

Partly

Are sufficient details of methods and analysis provided to allow replication by others?

No

Reviewer Expertise:

My areas of expertise include nuclear medicine, molecular imaging, PET/CT, oncologic imaging, diagnostic accuracy studies, and the clinical interpretation of imaging biomarkers.

I confirm that I have read this submission and believe that I have an appropriate level of expertise to confirm that it is of an acceptable scientific standard.

# Reviewer report: Reviewer response for version 2

**DOI:** 10.5256/f1000research.200275.r486353

**Published:** 2026-06-03

**Authors:** Prabhjeet Singh

**Affiliations:** 1 Stanford University, Stanford, California, USA

Authors have addressed the comments and ready to accept. I approve of this manuscript

Is the work clearly and accurately presented and does it cite the current literature?

Yes

If applicable, is the statistical analysis and its interpretation appropriate?

Partly

Are all the source data underlying the results available to ensure full reproducibility?

Partly

Is the study design appropriate and is the work technically sound?

Yes

Are the conclusions drawn adequately supported by the results?

Yes

Are sufficient details of methods and analysis provided to allow replication by others?

Partly

Reviewer Expertise:

Cancer Spatial imaging, Immunotherapeutic evaluation, metastatic tumor burden, nanochemistry.

I confirm that I have read this submission and believe that I have an appropriate level of expertise to confirm that it is of an acceptable scientific standard.

# Reviewer report: Reviewer response for version 2

**DOI:** 10.5256/f1000research.200275.r486352

**Published:** 2026-05-26

**Authors:** Mehmet Tarık Tatoğlu

**Affiliations:** 1 Göztepe Prof. Dr. Süleyman Yalçin City Hospital, Istanbul, Turkey

The revised version has improved substantially and addresses most of my previous concerns. I appreciate the authors’ careful efforts to strengthen the manuscript. In particular, the expanded PET/CT methodology, clearer description of the reference standard and pathological definitions, correction of the Section 4.4 inconsistency, de-emphasis of the F1-score, acknowledgement of sample-size limitations, and moderation of the originality claim have all improved the paper meaningfully.

 At this stage, the manuscript appears close to final form. Only one minor point remains. The abstract conclusion still reads somewhat more strongly than the main text regarding the rule-out value of a negative PET result, so a small wording adjustment for consistency may be helpful.

 Overall, this is now a much improved manuscript, and I appreciate the authors’ thoughtful revisions. Subject to this final clarification, I maintain my recommendation of Approved with Reservations.

Is the work clearly and accurately presented and does it cite the current literature?

Partly

If applicable, is the statistical analysis and its interpretation appropriate?

Partly

Are all the source data underlying the results available to ensure full reproducibility?

Partly

Is the study design appropriate and is the work technically sound?

Partly

Are the conclusions drawn adequately supported by the results?

Partly

Are sufficient details of methods and analysis provided to allow replication by others?

No

Reviewer Expertise:

My areas of expertise include nuclear medicine, molecular imaging, PET/CT, oncologic imaging, diagnostic accuracy studies, and the clinical interpretation of imaging biomarkers.

I confirm that I have read this submission and believe that I have an appropriate level of expertise to confirm that it is of an acceptable scientific standard, however I have significant reservations, as outlined above.

# Response

**Published:** 2026-06-10

**Authors:** Sarath Chandu Yadlapalli

**Affiliations:** Surgical Oncology, Yenepoya Medical College Hospital, Mangaluru, Karnataka, India

We thank the reviewer for this valuable suggestion.The abstract conclusion has been revised to better align with the findings presented in the main text and to avoid overstating the rule-out value of a negative PET result. The revised wording reflects the moderate sensitivity and relatively high negative predictive value of PET while emphasizing the need for interpretation in the context of overall clinical assessment.

# Reviewer report: Reviewer response for version 1

**DOI:** 10.5256/f1000research.196199.r475609

**Published:** 2026-04-17

**Authors:** Mehmet Tarık Tatoğlu

**Affiliations:** 1 Göztepe Prof. Dr. Süleyman Yalçin City Hospital, Istanbul, Turkey

This study addresses a clinically relevant question, namely the diagnostic performance of PET/CT for lymph node assessment in breast cancer patients proceeding directly to upfront surgery. The use of a pathology-based reference standard is an important strength, and the treatment-pathway-specific focus adds potential value. However, the current version has several methodological and interpretive issues that should be addressed to improve the rigor and reproducibility of the work. I therefore recommend Approved with Reservations, subject to meaningful revision of the points outlined below.

 Major comments

 1. PET/CT methodology

 The PET/CT methodology is insufficiently described to allow replication or critical appraisal. The manuscript should report the tracer type and administered dose, uptake time, scanner model and acquisition parameters, reconstruction algorithm, and attenuation correction method. Equally important, the criteria used to define lymph node positivity on PET/CT should be stated explicitly, whether based on visual assessment, a fixed SUV threshold, or another metric. The authors should also clarify the number of readers involved, their level of experience, and whether image interpretation was performed with or without knowledge of the clinical and pathological findings. The absence of these details substantially limits reproducibility.

 2. Reference standard

 The reference standard requires more precise characterization. It is not stated whether nodal assessment was based on sentinel lymph node biopsy, complete axillary dissection, targeted nodal excision, or a combination of these procedures. Given that the extent of nodal sampling directly affects the apparent false-negative rate, this distinction is clinically important. The pathological definition of nodal positivity should also be provided, including whether isolated tumor cells and micrometastases were classified together with macrometastatic disease or analyzed separately.

 3. Overstatement of rule-out value

 The conclusions place disproportionate emphasis on the rule-out value of a negative PET result. With a sensitivity of 69.23% (95% CI: 48.27–85.67%) and 8 false-negative cases among 26 pathologically confirmed node-positive patients, a negative PET finding cannot reliably exclude nodal involvement. The moderate Cohen’s kappa of 0.51 further underscores the imperfect agreement between PET/CT and histopathology. The conclusions should therefore be reframed accordingly: PET/CT may provide useful supplementary preoperative information, but it should not be positioned as a substitute for standard axillary staging procedures.

 4. Internal inconsistency in Section 4.4

 Section 4.4 is titled “Why sensitivity exceeds specificity,” yet the reported results show the opposite: specificity (81.82%) exceeds sensitivity (69.23%). This factual inconsistency should be corrected, and the accompanying discussion revised accordingly.

 5. Cohort derivation and flow diagram

 The derivation of the analytic cohort requires clearer explanation. The flow diagram indicates that 1,415 patients were assessed for eligibility, of whom 1,201 were excluded, leaving 214 with available PET/CT and histopathology reports. Of these, 144 subsequently underwent neoadjuvant chemotherapy and were excluded, yielding the final cohort of 70. However, the specific reasons for the initial 1,201 exclusions are not broken down, and it remains unclear whether exclusion of neoadjuvant patients introduced any selection bias into the surgical cohort. A more detailed description of the eligibility, exclusion, and cohort derivation steps would improve transparency.

 6. Table 1: “Primary tumor SUVmax: Nil” and numerical inconsistency

 Table 1 includes a “Primary tumor SUVmax: Nil” category (n=45, 64.3%) that is not explained in the text and is difficult to interpret. It is unclear whether this denotes the absence of an FDG-avid primary tumor, a missing or unacquired measurement, or a technical classification. This category should be defined clearly. In addition, there appears to be a minor inconsistency between the Results text and Table 1 regarding molecular subtype frequencies (Luminal B: n=27 in the text versus n=29 in the table), which should be reconciled.

 7. Statistical reporting and clinical interpretation

 The statistical reporting is adequate at a descriptive level, but several aspects of the clinical interpretation warrant attention. The F1-score, while mathematically valid, has limited intuitive value for a clinical staging audience and receives more emphasis than its interpretive importance justifies. More clinically useful would be a focused discussion of the false-negative rate (30.77%), its implications for missed nodal disease, and how PET/CT findings should be integrated alongside standard axillary assessment rather than used in isolation. In addition, the manuscript does not provide a sample size justification or other rationale for the precision of the estimates; given the wide confidence intervals observed, particularly for sensitivity, the resulting statistical uncertainty should be acknowledged more explicitly as a limitation.

 Minor comments

 1. Terminology consistency

 The manuscript alternates between “PET CT” and “PET/CT”. A single form should be selected and applied consistently throughout.

 2. Language and grammar

 The manuscript would benefit from language editing for grammar and style in several places. This does not affect the scientific content but would improve readability.

 3. Figure 2

 Figure 2 adds limited value in its current form, given that Table 2 already conveys the key numerical results. It could either be developed into a more informative visualization or simplified substantially.

 4. “First study” claim

 The claim in Section 4.1 that this is the “first study” to specifically characterize PET diagnostic accuracy in a homogeneous upfront surgical cohort should be tempered or supported by a reproducible literature search, as prior original studies have already examined FDG-PET/CT for preoperative axillary staging in breast cancer [1–3].

 Summary

 This is a potentially useful single-institution diagnostic accuracy study on a clinically relevant topic. The treatment-pathway-specific design and pathologically confirmed reference standard are genuine strengths. However, the paper requires meaningful revision, particularly with respect to: (1) the description of PET/CT methodology and reader blinding; (2) clarification of the reference standard and extent of nodal sampling; (3) correction of the internal inconsistency in Section 4.4; and (4) moderation of the conclusions regarding the reliability of negative PET findings. Addressing these points would substantially strengthen the work.

 References:

 Reference 1

 Reference 2

 Reference 3

Is the work clearly and accurately presented and does it cite the current literature?

Partly

If applicable, is the statistical analysis and its interpretation appropriate?

Partly

Are all the source data underlying the results available to ensure full reproducibility?

Partly

Is the study design appropriate and is the work technically sound?

Partly

Are the conclusions drawn adequately supported by the results?

Partly

Are sufficient details of methods and analysis provided to allow replication by others?

No

Reviewer Expertise:

My areas of expertise include nuclear medicine, molecular imaging, PET/CT, oncologic imaging, diagnostic accuracy studies, and the clinical interpretation of imaging biomarkers.

I confirm that I have read this submission and believe that I have an appropriate level of expertise to confirm that it is of an acceptable scientific standard, however I have significant reservations, as outlined above.

## References

[1] : Comparison of the Diagnostic Value of FDG-PET/CT and Axillary Ultrasound for the Detection of Lymph Node Metastases in Breast Cancer Patients. *Acta Radiologica* .2012;53(10) : 10.1258/ar.2012.110635 1092-1098 10.1258/ar.2012.110635 23002144

[2] : Does F-18 FDG-PET/CT Have an Additional Impact on Axillary Approach in Early-Stage Breast Cancer?. *European Journal of Breast Health* .2024;20(1) : 10.4274/ejbh.galenos.2023.2023-10-6 45-51 10.4274/ejbh.galenos.2023.2023-10-6 38187104 PMC10765458

[3] : Axillary staging with 18F-FDG PET/CT in early breast cancer: impact of tumor subtypes. *Annals of Saudi Medicine* .2025;45(3) : 10.5144/0256-4947.2025.145 145-153 10.5144/0256-4947.2025.145 PMC1214519240482189

# Response

**Published:** 2026-04-23

**Authors:** Sarath Chandu Yadlapalli

**Affiliations:** Surgical Oncology, Yenepoya Medical College Hospital, Mangaluru, Karnataka, India

1.  PET/CT methodology

 The PET/CT methodology is insufficiently described to allow replication or critical appraisal. The manuscript should report the tracer type and administered dose, uptake time, scanner model and acquisition parameters, reconstruction algorithm, and attenuation correction method. Equally important, the criteria used to define lymph node positivity on PET/CT should be stated explicitly, whether based on visual assessment, a fixed SUV threshold, or another metric. The authors should also clarify the number of readers involved, their level of experience, and whether image interpretation was performed with or without knowledge of the clinical and pathological findings. The absence of these details substantially limits reproducibility.

 We have provided a comprehensive methodology section detailing the PET/CT protocol, including the tracer type and administered dose, uptake time, scanner model, acquisition parameters, reconstruction algorithm, and attenuation correction technique. Additionally, we have clearly defined the criteria for lymph node positivity, along with the number of interpreters, their level of experience, and the blinding methodology employed

 2. Reference standard

 The reference standard requires more precise characterization. It is not stated whether nodal assessment was based on sentinel lymph node biopsy, complete axillary dissection, targeted nodal excision, or a combination of these procedures. Given that the extent of nodal sampling directly affects the apparent false-negative rate, this distinction is clinically important. The pathological definition of nodal positivity should also be provided, including whether isolated tumor cells and micrometastases were classified together with macrometastatic disease or analyzed separately.

 As pointed out by the reviewer, we have explicitly detailed the lymph nodal assessment methodology along with the pathological criteria used to define nodal positivity. We have also included definitions addressing micrometastasis and isolated tumor cells

 3. Overstatement of rule-out value

 The conclusions place disproportionate emphasis on the rule-out value of a negative PET result. With a sensitivity of 69.23% (95% CI: 48.27–85.67%) and 8 false-negative cases among 26 pathologically confirmed node-positive patients, a negative PET finding cannot reliably exclude nodal involvement. The moderate Cohen’s kappa of 0.51 further underscores the imperfect agreement between PET/CT and histopathology. The conclusions should therefore be reframed accordingly: PET/CT may provide useful supplementary preoperative information, but it should not be positioned as a substitute for standard axillary staging procedures.

 We have revised the statement to ensure alignment with the conclusion, as outlined above.

 4.  Internal inconsistency in Section 4.4

 Section 4.4 is titled “Why sensitivity exceeds specificity,” yet the reported results show the opposite: specificity (81.82%) exceeds sensitivity (69.23%). This factual inconsistency should be corrected, and the accompanying discussion revised accordingly.

 We thank the reviewer for highlighting this error, as it was inadvertently stated the other way. The statement has now been corrected.

 5. Cohort derivation and flow diagram

 The derivation of the analytic cohort requires clearer explanation. The flow diagram indicates that 1,415 patients were assessed for eligibility, of whom 1,201 were excluded, leaving 214 with available PET/CT and histopathology reports. Of these, 144 subsequently underwent neoadjuvant chemotherapy and were excluded, yielding the final cohort of 70. However, the specific reasons for the initial 1,201 exclusions are not broken down, and it remains unclear whether exclusion of neoadjuvant patients introduced any selection bias into the surgical cohort. A more detailed description of the eligibility, exclusion, and cohort derivation steps would improve transparency.

 It is clearly indicated in the flowchart that reports for 1201 patients—either PET/CT or final histopathology—were unavailable.

 6. Table 1: “Primary tumor SUVmax: Nil” and numerical inconsistency

 Table 1 includes a “Primary tumor SUVmax: Nil” category (n=45, 64.3%) that is not explained in the text and is difficult to interpret. It is unclear whether this denotes the absence of an FDG-avid primary tumor, a missing or unacquired measurement, or a technical classification. This category should be defined clearly. In addition, there appears to be a minor inconsistency between the Results text and Table 1 regarding molecular subtype frequencies (Luminal B: n=27 in the text versus n=29 in the table), which should be reconciled.

 Table 1 presents the lymph nodal SUVmax rather than the primary tumor SUVmax. This represents a technical classification only, and no conclusions were drawn based on this parameter.

 7. Statistical reporting and clinical interpretation

 The statistical reporting is adequate at a descriptive level, but several aspects of the clinical interpretation warrant attention. The F1-score, while mathematically valid, has limited intuitive value for a clinical staging audience and receives more emphasis than its interpretive importance justifies. More clinically useful would be a focused discussion of the false-negative rate (30.77%), its implications for missed nodal disease, and how PET/CT findings should be integrated alongside standard axillary assessment rather than used in isolation. In addition, the manuscript does not provide a sample size justification or other rationale for the precision of the estimates; given the wide confidence intervals observed, particularly for sensitivity, the resulting statistical uncertainty should be acknowledged more explicitly as a limitation.

 We agree that the F1‑score has limited intuitive value for a clinical staging audience. We have therefore reframed it as a secondary composite metric in the Methods and Results and removed it from the list of key strengths, placing interpretive emphasis on sensitivity, specificity, predictive values, likelihood ratios, and false‑negative rate.

 We have expanded the Discussion to more explicitly address the observed false‑negative rate of 30.77%, its implications for missed nodal disease, and the need to use PET/CT as an adjunct to, rather than a replacement for, standard axillary assessment (clinical examination, ultrasound, and sentinel lymph node biopsy).

 We now acknowledge that no a priori sample size calculation was performed and that the relatively small sample, especially the number of node‑positive cases, leads to wide confidence intervals for some diagnostic estimates. This statistical uncertainty is now explicitly discussed as a limitation and the findings are presented as hypothesis‑generating

**Minor comments**

 1. Terminology consistency

 The manuscript alternates between “PET CT” and “PET/CT”. A single form should be selected and applied consistently throughout.

 Terminology has been standardised throughout the manuscript; we now use ‘PET/CT’ consistently in the text, tables, and figure legends, with the full term ‘positron emission tomography/computed tomography (PET/CT)’ defined at first mention

 2. Language and grammar

 The manuscript would benefit from language editing for grammar and style in several places. This does not affect the scientific content but would improve readability.

 In response to the reviewer’s suggestion, the manuscript has been professionally edited for English language and style to improve clarity and readability, without altering the underlying scientific content.

 3. Figure 2

 Figure 2 adds limited value in its current form, given that Table 2 already conveys the key numerical results. It could either be developed into a more informative visualization or simplified substantially.

 In response to the reviewer’s comment, Figure 2 has been simplified to a concise bar‑chart summary of sensitivity, specificity, predictive values, and accuracy, with Table 2 retained as the primary source for the full numerical results and confidence intervals

 4. “First study” claim

 The claim in Section 4.1 that this is the “first study” to specifically characterize PET diagnostic accuracy in a homogeneous upfront surgical cohort should be tempered or supported by a reproducible literature search, as prior original studies have already examined FDG-PET/CT for preoperative axillary staging in breast cancer [1–3].

 We thank the reviewer for highlighting earlier work on FDG‑PET/CT for preoperative axillary staging. We have removed the absolute ‘first study’ claim from Section 4.1 and now state that our study adds treatment‑pathway–specific data on PET/CT diagnostic accuracy in a homogeneous upfront surgery cohort, explicitly acknowledging prior FDG‑PET/CT axillary staging studies

# Reviewer report: Reviewer response for version 1

**DOI:** 10.5256/f1000research.196199.r465072

**Published:** 2026-03-25

**Authors:** Prabhjeet Singh

**Affiliations:** 1 Stanford University, Stanford, California, USA

Few minor Comments requires readdressal before final acceptance. 

1. The final paragraph of the Introduction would benefit with a clearer articulation of the study objective, as well as an overview of the study design and plan.
2. Under the methodology section, please provide a complete description of the protocol parameters to improve clarity and reproducibility. Specifically, include details on tracer type and dose, uptake time, imaging protocol, scanner/acquisition parameters, reconstruction and corrections, and the quantification methods (SUV definitions, ROIs/VOIs).
3. Under the Study Design section (2.1), please provide details on the study location in accordance with the journal guidelines.
4. Figure 2 requires formatting and presentation improvements for better clarity. Ensure high resolution, consistent font size, color scheme, a comprehensive legend, axis labels with units, and a caption that clearly explains all symbols and panels.
5. An additional PET imaging figure could be incorporated to better demonstrate the selectivity and specificity. Ensure the caption clearly explains how the figure supports selectivity and specificity and keep the imaging panels well labeled and interpretable.
6. To maintain consistency and clarity, please use uniform terminology throughout the manuscript. For example, avoid mixing 'PET CT' and 'PET/CT'; please select one form and apply it consistently.

Is the work clearly and accurately presented and does it cite the current literature?

Yes

If applicable, is the statistical analysis and its interpretation appropriate?

Partly

Are all the source data underlying the results available to ensure full reproducibility?

Partly

Is the study design appropriate and is the work technically sound?

Yes

Are the conclusions drawn adequately supported by the results?

Yes

Are sufficient details of methods and analysis provided to allow replication by others?

Partly

Reviewer Expertise:

Cancer Spatial imaging, Immunotherapeutic evaluation, metastatic tumor burden, nanochemistry.

I confirm that I have read this submission and believe that I have an appropriate level of expertise to confirm that it is of an acceptable scientific standard, however I have significant reservations, as outlined above.

# Response

**Published:** 2026-04-23

**Authors:** Sarath Chandu Yadlapalli

**Affiliations:** Surgical Oncology, Yenepoya Medical College Hospital, Mangaluru, Karnataka, India

1. The final paragraph of the Introduction would benefit with a clearer articulation of the study objective, as well as an overview of the study design and plan.                                              This has been addressed as per the reviewer’s comment
2. Under the methodology section, please provide a complete description of the protocol parameters to improve clarity and reproducibility. Specifically, include details on tracer type and dose, uptake time, imaging protocol, scanner/acquisition parameters, reconstruction and corrections, and the quantification methods (SUV definitions, ROIs/VOIs).                      We have included a comprehensive methodology section detailing the PET/CT protocol, encompassing the tracer type and administered dose, uptake time, scanner model, acquisition parameters, reconstruction algorithm, and the quantification methods.
3. Under the Study Design section (2.1), please provide details on the study location in accordance with the journal guidelines.                                                                                    This has been revised in accordance with the journal guidelines, as suggested by the reviewer. 
4. Figure 2 requires formatting and presentation improvements for better clarity. Ensure high resolution, consistent font size, color scheme, a comprehensive legend, axis labels with units, and a caption that clearly explains all symbols and panels.                                          We have provided the original Word file used to generate the bar chart. However, as the journal does not accept this format previously, a lower-resolution version was submitted instead
5. An additional PET imaging figure could be incorporated to better demonstrate the selectivity and specificity. Ensure the caption clearly explains how the figure supports selectivity and specificity and keep the imaging panels well labeled and interpretable.                                   We have added the PET/CT image.
6. To maintain consistency and clarity, please use uniform terminology throughout the manuscript. For example, avoid mixing 'PET CT' and 'PET/CT'; please select one form and apply it consistently.                                                                                                                  Terminology has been standardised throughout the manuscript; we now use ‘PET/CT’ consistently in the text, tables, and figure legends, with the full term ‘positron emission tomography/computed tomography (PET/CT)’ defined at first mention.
